# 

**Published:** 1910-01

**Authors:** 


Sixty-fifth Year.

VOL. LXV. JANUARY, 1910. No. 6

### BUFFALO MEDICAL JOURNAL

LIBRARY.
SURGEON GENERAL'S OFFICE
JAN 3 1910

Established 1845 by AUSTIN FLINT M. D.

EDITOR:

WILLIAM WARREN POTTER, M. D.

ASSISTANT EDITORS:

NELSON W. WILSON, M. D. JAMES WRIGHT PUTNAM, M. D.

ASSOCIATE EDITORS:

ERNEST WENDE, M. D. JOHN A. RAFTER, M. D.
JOHN PARMENTER, M. D. JOHN A. MILLER, Ph. D.
MAUD J. FRYE, M. D.

